# Examining the relationship between physical and sexual violence and psychosocial health in young people living with HIV in rural South Africa

**DOI:** 10.1002/jia2.25654

**Published:** 2020-12-19

**Authors:** Lindsey M Filiatreau, Danielle Giovenco, Rhian Twine, F Xavier Gómez‐Olivé, Kathleen Kahn, Nicole Haberland, Audrey Pettifor

**Affiliations:** ^1^ Department of Epidemiology Gillings School of Global Public Health University of North Carolina at Chapel Hill Chapel Hill NC USA; ^2^ MRC/Wits Rural Public Health and Health Transitions Research Unit (Agincourt) School of Public Health Faculty of Health Science University of the Witwatersrand Johannesburg South Africa; ^3^ Population Council New York NY USA

**Keywords:** Violence, youth living with HIV, sub‐Saharan Africa, HIV, mental health, social support

## Abstract

**Introduction:**

Experiences of violence during youth contravene young people’s rights and increase the risk of depression and poor human immunodeficiency virus (HIV) care outcomes among youth living with HIV (YLWH). Intervention targets for mitigating the negative psychosocial effects of violence are needed, particularly in areas like rural South Africa where violence remains pervasive and mental healthcare is limited. This study aims to quantify the associations between physical and sexual violence and depressive symptoms in YLWH in rural South Africa and explore the modification of these associations by key measures of psychosocial well‐being.

**Methods:**

We conducted a cross‐sectional survey among 362 YLWH ages 12 to 24 in rural South Africa to ascertain participants’ history of physical and sexual violence, current depressive symptoms (Center for Epidemiological Studies‐Depression Scale) and levels of social support (Medical Outcomes Social Support Scale), resilience (Conner‐Davidson Resilience Scale) and self‐esteem (Rosenberg Self‐Esteem Scale). Log‐binomial regression was used to estimate the association between history of physical or sexual violence and clinically meaningful depressive symptoms (scores ≥16). Effect measure modification by high versus low resilience, social support and self‐esteem was assessed using likelihood ratio tests (α = 0.20).

**Results:**

A total of 334 individuals with a median age of 21 (interquartile range: 16 to 23) were included in this analysis. Most participants were female (71.3%), single (81.4%) and attending school (53.0%). Ninety‐four participants (28.1%) reported a history of physical or sexual violence and 92 individuals (27.5%) had clinically meaningful depressive symptoms. Meaningful depressive symptoms were significantly higher among participants with a history of physical or sexual violence as compared to those with no history of violence (adjusted prevalence ratio: 2.01; 95% CI: 1.43, 2.83). However, this association was significantly modified by social support (*p* = 0.04) and self‐esteem (*p* = 0.02).

**Conclusions:**

In this setting, the prevalence of meaningful depressive symptoms was significantly higher among YLWH with a history of physical or sexual violence as compared to those without a history of violence. However, higher levels of self‐esteem or social support appeared to mitigate this association. Programmes to improve self‐esteem and social support for youth have the potential to minimize depressive symptoms in YLWH who have experienced physical or sexual violence.

## 1 Introduction

Violence against young people is a worldwide crisis with interpersonal violence commonly cited as a leading cause of death among youth in most parts of the world [1]. Global estimates suggest nearly 1.5 billion children under the age of 18 have experienced some form of violence or neglect within the past year [2]. In the World Health Organization’s Africa region, it is estimated that 50% of children ages 2 to 17 have experienced bullying, witnessed violence, or experienced physical, sexual or emotional violence within the past year [2]. In South Africa, 42.2% of school‐going youth ages 15 to 17 in a nationally representative survey reported prior experiences of neglect, or physical, sexual or emotional maltreatment [3].

There is mounting evidence illuminating the long‐term psychological effects of violence experienced during early life [1, 4]. In one South African study, young people who had experienced sexual abuse were over two times as likely to report symptoms of anxiety and depression than those without experiences of sexual abuse [3]. Hsiao et al. also found that serious mental illness could be reduced by 10%, and self‐harm by 23%, if physical violence against children was prevented [5]. Poor mental health is a leading cause of health‐related disability among youth worldwide and should be prioritized to improve individuals’ long‐term health outcomes [6].

Youth and adolescents living with human immunodeficiency virus (HIV) are at an even higher risk of violence and poor psychological outcomes than their peers [7]. HIV‐related stigma and discrimination, the stress of navigating complex HIV care systems and the intra‐ and interpersonal difficulties surrounding disclosure of one’s HIV status, can compound the emotional difficulties characteristic of this period in the life course [8, 9, 10]. Experienced violence and poor mental health can subsequently lead to poor HIV care outcomes (e.g. antiretroviral treatment (ART) non‐adherence) [4, 11, 12, 13, 14, 15, 16]. In a study of 1060 South African youth living with HIV (YLWH), physical abuse by caregivers, witnessing domestic violence, teacher violence and verbal victimization by healthcare staff were all independently associated with ART non‐adherence [11]. Murphy et al. (2005) estimated that the hazard of treatment non‐adherence among depressed individuals was 2.21 times that of non‐depressed individuals [15].

Despite recent efforts to quantify the magnitude and detrimental effects of violence against youth, these efforts have not garnered the political or financial commitments necessary to fast‐track the elimination of the issue in South Africa. As a result, other avenues of mitigating violence against youth, and the poor psychological outcomes stemming from experienced violence, must be explored. Social support, resilience and self‐esteem have been found to improve mental health [9, 17, 18, 19, 20, 21, 22] and HIV care outcomes [23, 24, 25, 26, 27]. These domains could serve as potential intervention targets for mitigating the negative psychological effects of violence in YLWH.

This study aims to quantify the prevalence of experienced physical and sexual violence and clinically meaningful depressive symptoms in YLWH in rural South Africa. We also estimate the association between history of physical and sexual violence and current depressive symptoms and assess the modification of these estimates by social support, resilience and self‐esteem. Our goal is to identify potential intervention targets that can aid in mitigating the negative psychological effects of violence against YLWH in this context.

## 2 Methods

### 2.1 Setting

This study was conducted in the Agincourt Health and Socio‐Demographic Surveillance System (HDSS) study area located in rural Mpumalanga Province, South Africa, approximately 500 km northeast of Johannesburg [28]. The research area is home to over 120 000 individuals and is characterized by high levels of poverty [29], intimate partner violence [30], migration [31] and HIV [32]. Over half of 20‐year‐olds living within the study area are still enrolled in primary or secondary school, and few local post‐school economic opportunities exist, yielding high proportions of unemployment and migrant laborers in young adults [33, 34]. Access to public sector services and mental healthcare is severely limited [28], with almost no differentiated care for youth specifically.

### 2.2 Study population

We aimed to recruit all YLWH ages 12 to 24 who had a documented HIV‐positive test result in either: (1) Tier.net, South Africa’s national electronic HIV treatment and care register [35]; or, (2) Clinic Link, the HDSS clinic system created to link medical record data from patients in nine publicly funded area healthcare facilities to HDSS census data [36] for study participation. To maximize enrollment, participant recruitment was completed by study research assistants, area healthcare providers and area home‐based care providers who regularly interact with in and out of care YLWH in their respective coverage areas. Individuals were considered ineligible if they: (1) were pregnant; (2) received most of their HIV care outside the nine publicly funded healthcare facilities in the HDSS; (3) were enrolled in any other HIV treatment or care studies or (4) were outside the ages of 12 to 24 at enrollment. All eligibility criteria were determined through participant self‐report.

### 2.3 Study design and data collection

We conducted a cross‐sectional survey between April 2019 and August 2019 to ascertain the healthcare‐seeking experiences, psychosocial well‐being and history of violence of YLWH in the study area. The survey was administered by locally trained research assistants resident to the study site and fluent in English and Xitsonga – the local language. Surveys were conducted in a private location near the participants' residence in the participants' language of choice. Responses were recorded electronically on study tablets by the research assistants. Questions regarding participants’ experiences with violence were self‐administered using audio‐assisted self‐interview technology to minimize social desirability bias often induced when asking sensitive questions.

### 2.4 Measures

#### 2.4.1 Violence

Participants’ experiences with physical and sexual violence were captured using items developed by the World Health Organization [37]. Though this instrument was designed to assess experiences of violence in women, it has since been utilized among adolescents and men in sub‐Saharan Africa [37, 38, 39, 40, 41, 42]. Using this tool, participants were asked to indicate if a partner, family member or friend had ever slapped or thrown things at them, pushed or shoved them, hit them, kicked, dragged or beat them, choked or burnt them, or used or threatened to use a gun, knife or other weapon against them. Individuals were also asked to indicate if such violence had occurred within their lifetime and the prior 12 months, and how often it had occurred within the prior 12 months.

Sexual violence data were captured by asking participants if anyone had physically forced them to have sexual intercourse when they did not want to and if they had had sexual intercourse that they did not want because they were afraid of what the other person might do. Participants were asked to indicate if either of these events had occurred within their lifetime and the prior 12 months, what their relationship was with perpetrators of any reported sexual violence, and how often the violence occurred within the prior 12 months.

Previous experiences with physical violence alone, sexual violence alone, physical or sexual violence and experiences with both physical and sexual violence were considered. The composite outcome (any physical or sexual violence) was the primary outcome of interest.

#### 2.4.2 Depressive symptoms

Depressive symptoms were measured using the 20‐item Center for Epidemiological Studies‐Depression Scale (CES‐D) [43, 44], which has been previously validated in South African adolescents [45]. Cronbach’s alpha for this study was 0.76. Possible scores range from 0 to 60 with scores of 16 or higher indicative of clinically meaningful depressive symptoms [46].

#### 2.4.3 Social support

Social support was captured using a modified 8‐item Medical Outcomes Social Support Survey [47]. Versions of this scale have been used among South African YLWH with high reliability (α = 0.85) [48]. Cronbach’s alpha for this study was 0.92. Possible scores range from 8 to 40 with higher scores indicative of greater social support. Scores were dichotomized at the median to represent those with higher versus lower social support, given there is no standard cut point.

#### 2.4.4 Resilience

Resilience was captured using the 25‐item Conner‐Davidson Resilience Scale (CD‐RISC) [49], which has been used in South African adolescents with high reliability (α = 0.93) [50]. Cronbach’s alpha for this study was 0.93. Possible scores range from 0 to 100 with higher scores indicative of greater resilience. Scores were dichotomized at the median to represent those with higher versus lower resilience, given there is no standard cut point.

#### 2.4.5 Self‐esteem

Self‐esteem was captured using the 10‐item Rosenburg Self‐Esteem Scale [51], which has been used throughout South Africa with moderate to high reliability (α ranging from 0.78 to 0.94) [52, 53, 54, 55]. Cronbach’s alpha for this study was 0.77. Possible scores range from 0 to 30 with higher scores indicative of greater self‐esteem [51]. Scores were dichotomized at the median to represent those with higher versus lower self‐esteem, given there is no standard cut point.

#### 2.4.6 Other covariates

Specific covariates of interest included age (continuous), self‐reported gender (male/female/transgender/gender‐fluid) and relationship status (single/partnered). These were determined a priori and captured through self‐report.

### 2.5 Ethical review and informed consent

To be eligible for study participation, participants 18 and older were required to provide written informed consent. Those under the age of 18 were required to provide written assent and obtain written informed consent from a parent or guardian at least 18 years of age. Ethical approval for this study was obtained from the University of North Carolina at Chapel Hill’s Institutional Review Board, the University of the Witwatersrand’s Human Research Ethics Committee and Mpumalanga Provinces’ Provincial Health Research Committee. Clinic and community access was facilitated through the MRC/Wits‐Agincourt Unit’s Public Engagement Office.

### 2.6 Analysis

Descriptive statistics (counts/proportions or medians/interquartile ranges (IQR)) were utilized to characterize the study population overall and by history of physical or sexual violence. Key demographic characteristics of those included versus excluded and with and without a history of violence were compared using chi‐square tests and Wilcoxon‐rank sum tests. To account for missingness of individual scale items, missing items were imputed using the mean of an individual’s non‐missing scale items, given less than 30% of items were missing [56].

In our primary analyses, eight log‐binomial regression models were run to estimate the unadjusted and adjusted associations between (1) prior history of physical violence; (2) prior history of sexual violence; (3) prior history of physical or sexual violence and 4) prior history of both physical and sexual violence, and clinically meaningful depressive symptoms. Adjusted analyses controlled for age [57, 58], self‐reported gender [57, 58] and relationship status [59, 60, 61], which comprised the minimally sufficient adjustment set in the directed acyclic graph.

In our secondary analyses, the modification of the main estimate of association between history of physical or sexual violence and depressive symptoms by high or low levels of (1) social support, (2) self‐esteem and (3) resilience was assessed using three additional models which each included an interaction term between the exposure and modifier of interest. Likelihood ratio tests were used to assess meaningful effect measure modification at α = 0.20.

## 3 Results

A total of 362 individuals participated in this study, with 28 (7.7%) excluded from the analysis because of missing violence data. There were no significant differences in participants who were included versus excluded. Among included individuals, most self‐identified as female (71.3%), were aged 18 or older (70.7%), single (81.4%) and attending school (53.0%). Among those who were not attending school, most were unemployed (84.7%). About half of the respondents (50.3%) were orphans (Table 1).

**Table 1:** Key characteristics of 334 young people living with HIV in the Agincourt health and socio‐demographic surveillance system site in Mpumalanga Province, South Africa

	Total n(%)/ (n = 334)	No history of violence n(%)/ (n = 240)	History of violence n(%)/ (n = 94)	*p*‐value
Age (median, IQR)	21 (16 to 23)	20 (15.5 to 23)	21 (19 to 23)	0.15
Self‐reported gender				
Male	96 (28.7)	73 (30.4)	23 (24.5)	0.28
Female	238 (71.3)	167 (69.6)	71 (75.5)	
Education				
None/some primary	67 (20.1)	52 (21.7)	15 (16.0)	0.04
Completed primary	155 (46.4)	101 (42.1)	54 (57.4)	
Completed secondary+	112 (33.5)	87 (36.2)	25 (26.6)	
Orphanhood status				
Non‐orphan	166 (49.7)	119 (49.6)	47 (50.0)	0.77
Single	122 (36.5)	86 (35.8)	36 (38.3)	
Double	46 (13.8)	35 (14.6)	11 (11.7)	
Relationship status				
Single	272 (81.4)	207 (86.2)	65 (69.2)	<0.00
Partnered	62 (18.6)	33 (13.8)	29 (30.8)	
Depressive symptoms				
Non‐meaningful symptoms (<16)	242 (72.5)	189 (78.8)	53 (56.4)	<0.00
Meaningful symptoms (16+)	92 (27.5)	51 (21.2)	41 (43.6)	
Social support				
Low	153 (45.8)	104 (43.3)	49 (52.1)	0.15
High	181 (54.2)	136 (56.7)	45 (47.9)	
Resilience				
Low	160 (47.9)	109 (45.4)	51 (54.3)	0.15
High	174 (52.1)	131 (54.6)	43 (45.7)	
Self‐esteem				
Low	164 (49.1)	105 (43.8)	59 (62.8)	<0.00
High	170 (50.9)	135 (56.2)	35 (37.2)	

Ninety‐two individuals (27.5%) had meaningful depressive symptoms (Table 1). Median scores on the Medical Outcomes Social Support Scale, Conner‐Davidson Resilience Scale and Rosenberg Self‐Esteem scale were 38 (IQR: 32 to 40), 73 (IQR: 65 to 81) and 21 (IQR: 18 to 24) respectively. No significant differences were observed in the proportion of males and females reporting meaningful depressive symptoms (*p* = 0.49), higher self‐esteem (*p* = 0.45), higher resilience (*p* = 0.62) or higher social support (*p* = 0.15).

Ninety‐four participants (28.1%) reported at least one experience of physical or sexual violence within their lifetime. Of these individuals, 58 (61.7%) reported a history of physical violence only, 14 (14.9%) of sexual violence only and 22 (23.4%) of both types of violence (Table 2). Of the 80 individuals reporting a history of physical violence, 53 (66.3%) and 56 (70.0%) participants reported perpetration of violence by partners and family members respectively. Of the 36 individuals reporting a history of sexual violence, 11 (30.6%), 4 (11.1%) and 5 (13.9%) individuals reported being forced to have sex by a partner, family member or another individual respectively. Eighteen (50.0%) individuals reported having sex with someone (relationship unspecified) because they were afraid of what the person might do. Fifty‐nine individuals (17.7%) reported physical or sexual violence within the prior 12 months. No significant differences were observed between those with a history of violence by self‐reported gender (Table 1). However, partnered individuals, those who had completed primary school, those with low self‐esteem and those with depressive symptoms were more likely to have experienced violence than their counterparts (Table 1).

**Table 2:** Estimates of association between each type of violence and depressive symptoms

	Total N (col %)	Depressive symptoms N (row %)	uPR (95%CI)	aPR (95% CI)^a^
Physical violence				
No history	254 (76.0)	56 (22.1)	Ref	Ref
Some history	80 (24.0)	36 (45.0)	2.04 (1.46, 2.85)	2.02 (1.43, 2.84)
Sexual violence				
No history	298 (89.2)	72 (24.2)	Ref	Ref
Some history	36 (10.8)	20 (55.6)	2.30 (1.61, 3.28)	2.25 (1.58, 3.19)
Physical or sexual violence				
No history	240 (71.9)	51 (21.2)	Ref	Ref
Some history	94 (28.1)	41 (43.6)	2.05 (1.47, 2.87)	2.01 (1.43, 2.83)
Physical & sexual violence				
No history	312 (93.4)	77 (24.7)	Ref	Ref
Some history	22 (6.6)	15 (68.2)	2.76 (1.96, 3.90)	3.01 (2.06, 4.39)

aPR: adjusted prevalence ratio; CI: confidence interval; uPR, unadjusted prevalence ratio.

^a^ Adjusted for age, self‐reported gender and relationship status.

The prevalence of clinically meaningful depressive symptoms was significantly higher among those with a history of physical violence, sexual violence, physical or sexual violence and both physical and sexual violence as compared to those without a history of each type of violence (Table 2). The prevalence of depressive symptoms was highest in individuals who had experienced both physical and sexual violence (Table 2).

Social support and self‐esteem significantly modified the association between history of physical or sexual violence and clinically meaningful depressive symptoms (Table 3). Among individuals with low social support, those with a history of physical or sexual violence were more likely to report meaningful depressive symptoms than those with no history of physical or sexual violence (adjusted prevalence ratio (aPR)=2.44; 95% confidence interval (CI): 1.66, 3.59). However, no significant association was observed between a history of physical or sexual violence and depressive symptoms among individuals with high levels of social support (aPR: 1.09; 95% CI: 0.56, 2.17). Among individuals with low self‐esteem, those with a prior history of physical or sexual violence were more likely to report meaningful depressive symptoms than those with no history of physical or sexual violence (aPR = 2.50; 95% CI: 1.63, 3.83). However, no significant association was observed between a history of physical or sexual violence and depressive symptoms among individuals with high levels of self‐esteem (aPR: 1.03; 95% CI: 0.51, 2.07). There was no evidence of effect measure modification by resilience (Table 3).

**Table 3:** Modification of the associations between history of physical or sexual violence and depressive symptoms by social support, self‐esteem and resilience

Effect measure modifier	Value	aPR (95% CI)^a^	LRT *p*‐value
Social support (Medical Outcomes Social Support Scale)	High (scores ≥ 38)	1.09 (0.56, 2.17)	0.04
	Low (scores < 38)	2.44 (1.66, 3.59)	
Self‐esteem (Rosenberg Self Esteem Scale)	High (scores ≥ 21)	1.03 (0.51, 2.07)	0.02
	Low (scores < 21)	2.50 (1.63, 3.83)	
Resilience (Conner‐Davidson Resilience Scale)	High (scores ≥ 73)	1.67 (0.96, 2.92)	0.46
	Low (scores < 73)	2.17 (1.41, 3.34)	

aPR, adjusted prevalence ratio; CI, confidence interval; LRT, likelihood ratio test.

^a^ Adjusted for age, self‐reported gender, relationship status, the modifier of interest and an interaction term between the modifier and history of violence.

## 4 Discussion

This study estimated the association between physical and sexual violence and clinically meaningful depressive symptoms among YLWH in rural South Africa. To our knowledge, it is the first study to assess the modification of this association by social support, resilience and self‐esteem. Our results highlight the fact that improved self‐esteem and social support systems could mitigate the negative psychological effects of violence.

Depressive symptoms were common among study respondents with over 25% of participants reporting clinically meaningful symptoms. Recent studies of mental health among South African and Malawian YLWH estimated that just 8% and 19% of participants were depressed respectively [9, 62]. Although these studies used different depression measures, and caution should be taken when drawing direct comparisons, our results remain of consequence, highlighting the unmet need for mental healthcare within the HDSS. Given the resource constraints of the study area, it is unlikely that clinic‐based mental healthcare and pharmacological treatment will sufficiently improve to meet the needs of resident YLWH in the near future. Less resource‐intensive interventions that incorporate lay counsellors, peer support groups and community action teams, should be implemented as they are both feasible and effective at improving mental health in YLWH [63, 64].

While national estimates suggest 18% of individuals have experienced physical violence by the age of 18, 24% of study participants reported experiences of physical violence [3, 65]. This heightened estimate was expected, given a majority of study participants were 18 or older. On the other hand, national estimates of sexual violence are similar to study estimates at 12% [65] and 11% respectively. Consistent with the literature, partnered individuals and those who had completed primary school were more likely to report a history of violence than single individuals and those with more or less schooling [66]. Violence prevention efforts should target these individuals most‐at‐risk.

Our results indicate the prevalence of meaningful depressive symptoms is significantly higher in individuals with a history of physical or sexual violence when compared with those without a history of violence. The prevalence of depressive symptoms was highest in individuals with a history of both physical and sexual violence. These results are similar to study findings in a range of settings [67, 68, 69, 70] and suggest that minimizing physical and sexual violence against youth could decrease depressive symptoms later in life. Unfortunately, interventions aimed at minimizing violence against youth, and the consequences of violence (e.g. economic interventions, parental support and coaching interventions, gender and violence norms interventions, etc.) have yielded mixed effects [71, 72, 73, 74, 75, 76, 77]. While the Responsible, Engaged and Loving Fathers intervention decreased perpetration of physical violence against children in Uganda [75], and the Common Elements Treatment Approach intervention decreased intimate partner violence against young women in Zambia [76], the Creating Opportunities through Mentorship, Parental Involvement and Safe Spaces caregiver training had no effect on violence against adolescent girls and young women in the Democratic Republic of the Congo [77].

Results from our modification assessment suggest the association between physical or sexual violence and depressive symptoms is mitigated in individuals with higher social support or self‐esteem. Although a number of interventions aimed at improving social support or self‐esteem among South African YLWH have proven ineffective, interventions from similar settings have demonstrated success [78, 79]. The Zvandiri programme, which began as a single support group in Zambia, aims to ensure the physical, social and mental well‐being of YLWH to improve HIV care outcomes. Since its inception, this programme has evolved to offer clinic and community‐based health services and peer psychosocial support for YLWH, significantly improving psychosocial and HIV care outcomes among participants [25, 80]. The Family Strengthening intervention, which aims to improve parenting skills, increase family connectedness, provide psychoeducation and strengthen social support, has also improved psychosocial outcomes in HIV‐affected Rwandese youth [81]. With the scale‐up of evidence‐based interventions that strengthen young people’s social support networks and self‐esteem, it is possible to minimize depressive symptoms associated with experiences of physical or sexual violence in YLWH.

This study makes significant contributions to the literature on YLWH. However, there are limitations. We aimed to interview all YLWH ages 12 to 24 in the HDSS who were not pregnant and met additional study inclusion criteria. Despite our multi‐pronged recruitment approach, a number of eligible individuals, likely those out of care and most vulnerable to violence and poor psychological outcomes, could not be located or refused to participate. Pregnant women, who are at heightened risk of violence [82, 83], were excluded at the request of the ethical review boards. As a result of these study population limitations, our results may underestimate the prevalence of violence and depressive symptoms among YLWH overall. Generalizability of study findings should be limited to non‐pregnant YLWH in similar settings who were linked to care after diagnosis. Given the cross‐sectional nature of the study, recall bias, whereby participants with depressive symptoms are more likely to recall experiences with violence, is a threat and could bias our results away from the null [84]. Self‐interview technology was utilized to reduce under‐reporting of experienced violence. However, participants fearing a breach in confidentiality of study data may still have under‐reported experienced violence, particularly if they felt vulnerable to subsequent violence. Finally, these results are not directly comparable to studies of violence against children, as the majority of participants were 18 or older and may have reported experiences of violence occurring in young adulthood.

Despite these challenges, our method of participant recruitment allowed us to interact with YLWH who were both in and out of HIV care. In addition, the inclusion of participants ages 12 to 24 allowed us to gain perspectives of YLWH as they navigate the difficult transition from adolescence into young adulthood. More broadly, this study quantifies the magnitude of violence against YLWH and characterizes the psychosocial well‐being of a hard‐to‐reach population in a resource‐constrained setting where increased access to adequate care and social programming is crucial to upholding the rights of and improving the long‐term health of this population.

Future studies should aim to recruit more YLWH and actively engage with individuals from the point of diagnosis in order to characterize the effects of violence over time and identify opportunities to intervene. Low resource interventions that are proven to improve the self‐esteem, social support and overall emotional well‐being of YLWH, such as the Zvandiri programme, should be implemented, tested for efficacy, and scaled up accordingly. It is imperative that we act to meet the mental healthcare needs of young people, and uphold their rights through violence prevention.

## 5 Conclusions

Physical and sexual violence persists among YLWH in the Agincourt HDSS, significantly contributing to depressive symptoms within this population. Efforts to minimize the occurrence of physical and sexual violence against youth should be implemented. Moreover, we found that the association between violence and depressive symptoms was mitigated in those with high self‐esteem or social support. Efforts to reduce violence against youth should be coupled with interventions that aim to improve social support networks and the self‐esteem of young people in order to achieve the maximum reduction in depressive symptoms among YLWH in this setting.

## Competing Interests

All authors declare that they have no competing interests.

## Authors’ Contributions

The author LF and AP developed the survey questionnaire. The author LF designed the survey in Survey Solutions, coordinated overall data collection, performed the data analysis and wrote the initial draft of the manuscript. The authors DG and AP provided support during data collection and analysis activities. The authors RT, KK and XGO provided onsite support throughout data collection activities and facilitated community access for research activities. The authors LF, DG, NH, RT, XGO, KK and AP contributed to the interpretation of study findings and manuscript revisions, and read and approved the final manuscript.

## References

[1] Mercy JA , Hillis SD , Butchart A , Bellis MA , Ward CL , Fang X , et al. Disease Control Priorities. 3rd ed. Washington, DC: The World Bank; 2017. Volume 7: Injury Prevention and Environmental Health.

[2] Hillis S , Mercy J , Amobi A , Kress H . Global prevalence of past‐year violence against children: A systematic review and minimum estimates. Pediatrics. 2016;137:e20154079. 26810785 10.1542/peds.2015-4079 PMC6496958

[3] Artz L , Burton P , Ward C , Leoschur L , Phyfer J , Lloyd S , et al. Optimus Study South Africa: Technical Report. Zurich, Switzerland: UBS Optimus Foundation; 2016.

[4] Kidman R , Violari A , Kidman R . Dating violence against HIV‐infected youth in South Africa: Associations with sexual risk behavior, medication adherence, and mental health. J Acquir Immune Defic Syndr. 2018;77(1):64–71. 29040165 10.1097/QAI.0000000000001569 PMC5720896

[5] Hsiao C , Fry D , Ward CL , Ganz G , Casey T , Zheng X , et al. Violence against children in South Africa: The cost of inaction to society and the economy. BMJ Glob Heal. 2018;3:e000573. 10.1136/bmjgh-2017-000573 PMC5838395 29515918

[6] Kieling C , Baker‐Henningham H , Belfer M , Conti G , Ertem I , Omigbodun O , et al. Child and adolescent mental health worldwide: evidence for action. Lancet. 1901;2011(378):1515–40. 10.1016/S0140-6736(11)60827-1 22008427

[7] Vreeman RC , McCoy BM , Lee S . Mental health challenges among adolescents living with HIV. J Int AIDS Soc. 2017;20:21497. 28530045 10.7448/IAS.20.4.21497 PMC5577712

[8] Millstein S , Petersen A , Nightingale E . Promoting the health of adolescents: new directions for the twenty‐first century. New York: Oxford University Press Inc; 1994.

[9] West N , Schwartz S , Mudavanhu M , Hanrahan C , France H , Nel J , et al. Mental health in South African adolescents living with HIV. AIDS Care. 2019;31(1):117–24. 30304947 10.1080/09540121.2018.1533222

[10] Cheng Y , Li X , Lou C , Sonenstein FL , Kalamar A , Jejeebhoy S , et al. The association between social support and mental health among vulnerable adolescents in five cities: findings from the study of the well‐being of adolescents in vulnerable environments. J Adolesc Health. 2014;55(6):S31–8. 25454000 10.1016/j.jadohealth.2014.08.020

[11] Cluver L , Meinck F , Toska E , Orkin FM , Hodes R , Sherr L . Multitype violence exposures and adolescent antiretroviral nonadherence in South Africa. AIDS. 2018;32(8):975–83. 29547438 10.1097/QAD.0000000000001795 PMC6037279

[12] Kim MH , Mazenga AC , Yu X , Ahmed S , Paul ME , Kazembe PN , et al. High self‐reported non‐adherence to antiretroviral therapy amongst adolescents living with HIV in Malawi: Barriers and associated factors. J Int AIDS Soc. 2017;20:21437. 28406275 10.7448/IAS.20.1.21437 PMC5515061

[13] Kacanek D , Malee K , Mellins CA , Tassiopoulos K , Smith R , Grant M , et al. Exposure to violence and virologic and immunological outcomes among youth with perinatal HIV in the pediatric HIV/AIDS cohort study. J Adolesc Health. 2016;59(1):30–7. 27089837 10.1016/j.jadohealth.2016.03.004 PMC4920719

[14] Fawzi MCS , Ng L , Kanyanganzi F , Kirk C , Bizimana J , Cyamatare F , et al. Mental health and antiretroviral adherence among youth living with HIV in Rwanda. Pediatrics. 2016;138:e20153235. 27677570 10.1542/peds.2015-3235 PMC5051202

[15] Murphy DA , Belzer M , Durako SJ , Sarr M , Wilson CM , Muenz LR . Longitudinal antiretroviral adherence among adolescents infected with human immunodeficiency virus. Arch Pediatr Adolesc Med. 2005;159(8):764–770. 16061785 10.1001/archpedi.159.8.764

[16] Kuhns LM , Hotton AL , Garofalo R , Muldoon AL , Jaffe K , Bouris A , et al. An index of multiple psychosocial, syndemic conditions is associated with antiretroviral medication adherence among HIV‐positive youth. AIDS Patient Care St. 2016;30(4):185–92. 10.1089/apc.2015.0328 PMC4827312 27028184

[17] Hjemdal O , Vogel PA , Solem S , Hagen K , Stiles TC . The relationship between resilience and levels of anxiety, depression, and obsessive‐compulsive symptoms in adolescents. Clin Psychol Psychother. 2011;18(4):314–21. 20806419 10.1002/cpp.719

[18] Brage D , Meredith W . A causal model of adolescent depression. J Psychol Interdiscip Appl. 1994;128(4):455–68. 10.1080/00223980.1994.9712752 7932297

[19] Rosenberg M , Schooler C , Schoenbach C . Self‐esteem and adolescent problems: modeling reciprocal effects. Am Sociol Rev. 1989;54(6):1004.

[20] Orth U , Robins RW , Roberts BW . Low self‐esteem prospectively predicts depression in adolescence and young adulthood. J Pers Soc Psychol. 2008;95(3):695–708. 18729703 10.1037/0022-3514.95.3.695

[21] Hjemdal O , Aune T , Reinfjell T , Stiles TC , Friborg O . Resilience as a predictor of depressive symptoms: a correlational study with young adolescents. Clin Child Psychol Psychiatry. 2007;12(1):91–104. 17375811 10.1177/1359104507071062

[22] Dale SK , Weber KM , Cohen MH , Kelso GA , Cruise RC , Brody LR . Resilience moderates the association between childhood sexual abuse and depressive symptoms among women with and at‐risk for HIV. AIDS Behav. 2015;19(8):1379–87. 25085079 10.1007/s10461-014-0855-3 PMC4314513

[23] Damulira C , Mukasa MN , Byansi W , Nabunya P , Kivumbi A , Namatovu P , et al. Examining the relationship of social support and family cohesion on ART adherence among HIV‐positive adolescents in southern Uganda: baseline findings. Vulnerable Child Youth Stud. 2019;14(2):181–90. 31149021 10.1080/17450128.2019.1576960 PMC6538035

[24] Mavhu W , Berwick J , Chirawu P , Makamba M , Copas A , Dirawo J , et al. Enhancing psychosocial support for HIV positive adolescents in Harare, Zimbabwe. Cameron DW, editor. PLoS One. 2013;8:e70254. 23894625 10.1371/journal.pone.0070254 PMC3720910

[25] Willis N , Napei T , Armstrong A , Jackson H , Apollo T , Mushavi A , et al. Zvandiri‐bringing a differentiated service delivery program to scale for children, adolescents, and young people in Zimbabwe. J Acquir Immune Defic Syndr. 2018;78 Suppl 2:S115–23. 29994833 10.1097/QAI.0000000000001737

[26] Tyer‐Viola LA , Corless IB , Webel A , Reid P , Sullivan KM , Nichols P . Exploring predictors of medication adherence among HIV‐positive women in North America. J Obstet Gynecol Neonatal Nurs. 2014;43(2):168–78. 10.1111/1552-6909.12288 PMC4409428 24502460

[27] Katz IT , Bogart LM , Dietrich JJ , Leslie HH , Iyer HS , Leone D , et al. Understanding the role of resilience resources, antiretroviral therapy initiation, and HIV‐1 RNA suppression among people living with HIV in South Africa: a prospective cohort study. AIDS. 2019;33:S71–9. 31397725 10.1097/QAD.0000000000002175 PMC6712569

[28] Kahn K , Collinson MA , Xavier Gómez‐olivé F , Mokoena O , Twine R , Mee P , et al. Profile: Agincourt health and socio‐demographic surveillance system. Int J Epidemiol. 2012;41(4):988–1001. 22933647 10.1093/ije/dys115 PMC3429877

[29] Kabudula CW , Houle B , Collinson MA , Kahn K , Gómez‐Olivé FX , Tollman S , et al. Socioeconomic differences in mortality in the antiretroviral therapy era in Agincourt, rural South Africa, 2001–13: a population surveillance analysis. Lancet Glob Heal. 2017;5(9):e924–35. 10.1016/S2214-109X(17)30297-8 PMC5559644 28807190

[30] Selin A , DeLong SM , Julien A , MacPhail C , Twine R , Hughes JP , et al. Prevalence and associations, by age group, of IPV among AGYW in rural South Africa. SAGE Open. 2019;9:215824401983001. 10.1177/2158244019830016 PMC6697129 31423351

[31] Collinson MA , White MJ , Bocquier P , McGarvey ST , Afolabi SA , Clark SJ , et al. Migration and the epidemiological transition: Insights from the Agincourt sub‐district of northeast South Africa. Glob Health Action. 2014;7 SUPP.1:23514. 24848656 10.3402/gha.v7.23514 PMC4028907

[32] Gómez‐Olivé FX , Angotti N , Houle B , Klipstein‐Grobusch K , Kabudula C , Menken J , et al. Prevalence of HIV among those 15 and older in rural South Africa. AIDS Care. 2013;25(9):1122–8. 23311396 10.1080/09540121.2012.750710 PMC3778517

[33] Collinson MA . Striving against adversity: the dynamics of migration, health and poverty in rural South Africa. Glob Health Action. 2010;3:5080. 10.3402/gha.v3i0.5080 PMC2882287 20531981

[34] Collinson MA , White MJ , Ginsburg C , Gómez‐Olivé FX , Kahn K , Tollman S . Youth migration, livelihood prospects and demographic dividend: A comparison of the Census 2011 and Agincourt Health and Demographic Surveillance System in the rural northeast of South Africa. African Popul Stud. 2016;30:2629. 10.11564/30-2-852 PMC5486969 28663669

[35] Osler M , Hilderbrand K , Hennessey C , Arendse J , Goemaere E , Ford N , et al. A three‐tier framework for monitoring antiretroviral therapy in high HIV burden settings. J Int AIDS Soc. 2014;17:18908. 24780511 10.7448/IAS.17.1.18908 PMC4005043

[36] Kabudula CW , Clark BD , Gómez‐Olivé FX , Tollman S , Menken J , Reniers G . The promise of record linkage for assessing the uptake of health services in resource constrained settings: a pilot study from South Africa. BMC Med Res Methodol. 2014;14(1):71. 24884457 10.1186/1471-2288-14-71 PMC4041350

[37] Garcia‐Moreno C , Jansen HAFM , Ellsberg M , Heise L , Watts C . WHO multi‐country study on women’s health and domestic violence against women: Initial results on prevalence, health outcomes and women’s responses. Geneva, Switzerland: World Health Organization; 2005.

[38] George G , Cawood C , Puren A , Khanyile D , Gerritsen A , Govender K , et al. Evaluating DREAMS HIV prevention interventions targeting adolescent girls and young women in high HIV prevalence districts in South Africa: protocol for a cross‐sectional study. BMC Womens Health. 2020;20(1):7. 31948429 10.1186/s12905-019-0875-2 PMC6966796

[39] Orindi BO , Maina BW , Muuo SW , Birdthistle I , Carter DJ , Floyd S , et al. Experiences of violence among adolescent girls and young women in Nairobi’s informal settlements prior to scale‐up of the DREAMS Partnership: Prevalence, severity and predictors. Mavhu W, editor. PLoS One. 2020;15:e0231737. 32320405 10.1371/journal.pone.0231737 PMC7176122

[40] Nguyen N , Powers KA , Miller WC , Howard AG , Halpern CT , Hughes JP , et al. Sexual partner types and incident HIV infection among rural South African adolescent girls and young women enrolled in HPTN 068: a latent class analysis. J Acquir Immune Defic Syndr. 2019;82(1):24–33. 31169772 10.1097/QAI.0000000000002096 PMC6692200

[41] Nyato D , Materu J , Kuringe E , Zoungrana J , Mjungu D , Lemwayi R , et al. Prevalence and correlates of partner violence among adolescent girls and young women: Evidence from baseline data of a cluster randomised trial in Tanzania. Stark L, editor. PLoS One. 2019;14:e0222950. 31593577 10.1371/journal.pone.0222950 PMC6782098

[42] Mutiso VN , Musyimi CW , Gitonga I , Rebello TJ , Tele A , Pike KM , et al. Toward community coverage on self‐screening, diagnosis, and help‐seeking behavior for both gender victims of intimate partner violence (IPV) in a Kenyan setting: the development of IPV‐brief self‐screener (IPV‐BSS) version of the WHO‐IPV instrument. J Interpers Violence. 2019;886260519855666. 31208269 10.1177/0886260519855666

[43] Radloff LS . The CES‐D scale: a self‐report depression scale for research in the general population. Appl Psychol Meas. 1977;1(3):385–401.

[44] Radloff LS . The use of the Center for Epidemiologic Studies Depression Scale in adolescents and young adults. J Youth Adolesc. 1991;20(2):149–66. 24265004 10.1007/BF01537606

[45] Pretorius TB . Cross‐cultural application of the Center for Epidemiological Studies Depression Scale: a study of black South African students. Psychol Rep. 1991;69(3 Pt 2):1179–85. 1792288 10.2466/pr0.1991.69.3f.1179

[46] Weissman MM , Sholomskas D , Pottenger M , Prusoff BA , Locke BZ . Assessing depressive symptoms in five psychiatric populations: a validation study. Am J Epidemiol. 1977;106(3):203–14. 900119 10.1093/oxfordjournals.aje.a112455

[47] Moser A , Stuck AE , Silliman RA , Ganz PA , Clough‐Gorr KM . The eight‐item modified Medical Outcomes Study Social Support Survey: Psychometric evaluation showed excellent performance. J Clin Epidemiol. 2012;65(10):1107–16. 22818947 10.1016/j.jclinepi.2012.04.007 PMC4119888

[48] Casale M , Boyes M , Pantelic M , Toska E , Cluver L . Suicidal thoughts and behaviour among South African adolescents living with HIV: Can social support buffer the impact of stigma? J Affect Disord. 2019;245:82–90. 30368074 10.1016/j.jad.2018.10.102

[49] Connor K , Davidson J . Development of a new resilience scale: the Connor‐Davidson Resilience Scale (CD‐RISC). Clinical Trial. 2003;2(18):76–82. 10.1002/da.10113 12964174

[50] Jørgensen IE , Seedat S . Factor structure of the Connor‐Davidson Resilience Scale in South African adolescents. Int J Adolesc Med Health. 2008;20(1):23–32. 18540281

[51] Rosenberg M . Society and the adolescent self‐image. Princeton, NJ: Princeton University Press; 1965.

[52] Westaway MS , Wolmarans L . Depression and self‐esteem: rapid screening for depression in black, low literacy, hospitalized tuberculosis patients. Soc Sci Med. 1992;35(10):1311–5. 1439914 10.1016/0277-9536(92)90184-r

[53] Schmitt DP , Allik J . Simultaneous administration of the Rosenberg self‐esteem scale in 53 nations: exploring the universal and culture‐specific features of global self‐esteem. J Pers Soc Psychol. 2005;89(4):623–42. 16287423 10.1037/0022-3514.89.4.623

[54] Baranik LE , Meade AW , Lakey CE , Lance CE , Hu C , Hua W , et al. Examining the differential item functioning of the Rosenberg Self‐Esteem Scale across eight countries. J Appl Soc Psychol. 2008;38(7):1867–904.

[55] Westaway MS , Jordaan ER , Tsai J . Investigating the psychometric properties of the Rosenberg Self‐Esteem scale for South African residents of Greater Pretoria. Eval Health Prof. 2015;38(2):181–99. 24064430 10.1177/0163278713504214

[56] Shrive FM , Stuart H , Quan H , Ghali WA . Dealing with missing data in a multi‐question depression scale: a comparison of imputation methods. BMC Med Res Methodol. 2006;6(1):57. 17166270 10.1186/1471-2288-6-57 PMC1716168

[57] World Health Organization . Preventing child maltreatment: a guide to taking action and generating evidence. Geneva: Switzerland; 2006.

[58] Meinck F , Cluver LD , Boyes ME , Mhlongo EL . Risk and protective factors for physical and sexual abuse of children and adolescents in africa: a review and implications for practice. Trauma, Violence, Abus. 2015;16(1):81–107. 10.1177/1524838014523336 24648489

[59] Tenkorang EY , Asamoah‐Boaheng M , Owusu AY . Intimate partner violence (IPV) against HIV‐positive women in sub‐Saharan Africa: A Mixed‐Method Systematic Review and Meta‐Analysis. Trauma, Violence, Abus. 2020;152483802090656. 10.1177/1524838020906560 32067599

[60] Bulloch AG , Williams JV , Lavorato DH , Patten SB . The relationship between major depression and marital disruption is bidirectional. Depress Anxiety. 2009;26(12):1172–7. 19798680 10.1002/da.20618

[61] Maman S , Groves AK , Mcnaughton Reyes HL , Moodley D . Diagnosis and disclosure of HIV status: Implications for women’s risk of physical partner violence in the postpartum period. J Acquir Immune Defic Syndr. 2016;72(5):546–51. 27028499 10.1097/QAI.0000000000001012 PMC4942348

[62] Kim MH , Mazenga AC , Yu X , Devandra A , Nguyen C , Ahmed S , et al. Factors associated with depression among adolescents living with HIV in Malawi. BMC Psychiatry. 2015;15(1):264. 26503291 10.1186/s12888-015-0649-9 PMC4624356

[63] Osborn TL , Wasil AR , Venturo‐Conerly KE , Schleider JL , Weisz JR . Group intervention for adolescent anxiety and depression: outcomes of a randomized trial with adolescents in Kenya. Behav Ther. 2020;51(4):601–15. 32586433 10.1016/j.beth.2019.09.005

[64] Lovero KL , Giusto AM , Wainberg ML . Evidence for efficacy of psychosocial interventions in LMICs. Lancet Psychiatry. 2020;7(2):113–14. 31948936 10.1016/S2215-0366(19)30531-0 PMC7330888

[65] Ward CL , Artz L , Leoschut L , Kassanjee R , Burton P . Sexual violence against children in South Africa: a nationally representative cross‐sectional study of prevalence and correlates. Lancet Glob Health. 2018;6(4):e460–68. 10.1016/S2214-109X(18)30060-3 29530424

[66] Akamike IC , Uneke CJ , Uro‐Chukwu HC , Okedo‐Alex IN , Chukwu OE . Predictors and facilitators of gender‐based violence in sub‐Saharan Africa: a rapid review. J Glob Health Rep. 2019;3:e2019076.

[67] Barrios YV , Gelaye B , Zhong Q , Nicolaidis C , Rondon MB , Garcia PJ , et al. Association of Childhood physical and sexual abuse with intimate partner violence, poor general health and depressive symptoms among pregnant women. Elhai JD, editor. PLoS One. 2015;10:e0116609. 25635902 10.1371/journal.pone.0116609 PMC4312043

[68] Devries KM , Mak JY , Bacchus LJ , Child JC , Falder G , Petzold M , et al. Intimate partner violence and incident depressive symptoms and suicide attempts: a systematic review of longitudinal studies. Tsai AC, editor. PLoS Medicine. 2013;10:e1001439. 23671407 10.1371/journal.pmed.1001439 PMC3646718

[69] Dubé C , Gagné MH , Clément MÈ , Chamberland C . Community violence and associated psychological problems among adolescents in the general population. J Child Adolesc Trauma. 2018;11(4):411–20. 30546818 10.1007/s40653-018-0218-8 PMC6267123

[70] Weiss EL , Longhurst JG , Mazure CM . Special articles childhood sexual abuse as a risk factor for depression in women: psychosocial and neurobiological correlates. Am J Psychiatry. 1999;156(6):816–28. 10360118 10.1176/ajp.156.6.816

[71] MacMillan HL , Wathen CN , Barlow J , Fergusson DM , Leventhal JM , Taussig HN . Interventions to prevent child maltreatment and associated impairment. Lancet. 2009;373(9659):250–66. 19056113 10.1016/S0140-6736(08)61708-0

[72] Bacchus LJ , Colombini M , Contreras Urbina M , Howarth E , Gardner F , Annan J , et al. Exploring opportunities for coordinated responses to intimate partner violence and child maltreatment in low and middle income countries: a scoping review. Psychol Health Med. 2017;22:135–65. 28150500 10.1080/13548506.2016.1274410

[73] Righi MK , Orchowski LM , Kuo C . Integrated Intimate Partner Violence and Human Immunodeficiency Virus Interventions in Sub‐Saharan Africa: A Systematic Review Targeting or including Adolescents. Violence Gend. 2019;6(2):92–104. 31297395 10.1089/vio.2018.0027 PMC6602102

[74] Mannell J , Willan S , Shahmanesh M , Seeley J , Sherr L , Gibbs A . Why interventions to prevent intimate partner violence and HIV have failed young women in southern Africa. J Int AIDS Soc. 2019;22:e25380. 31441229 10.1002/jia2.25380 PMC6706780

[75] Ashburn K , Kerner B , Ojamuge D , Lundgren R . Evaluation of the responsible, engaged, and loving (REAL) fathers initiative on physical child punishment and intimate partner violence in Northern Uganda. Prev Sci. 2017;18(7):854–64. 27738782 10.1007/s11121-016-0713-9 PMC5602091

[76] Murray LK , Kane JC , Glass N , van Wyk SS , Melendez F , Paul R , et al. Effectiveness of the Common Elements Treatment Approach (CETA) in reducing intimate partner violence and hazardous alcohol use in Zambia (VATU): A randomized controlled trial. PLoS Medicine. 2020;17:e1003056. 32302308 10.1371/journal.pmed.1003056 PMC7164585

[77] Stark L , Seff I , Asghar K , Roth D , Bakamore T , MacRae M , et al. Building caregivers’ emotional, parental and social support skills to prevent violence against adolescent girls: Findings from a cluster randomised controlled trial in Democratic Republic of Congo. BMJ Glob Health. 2018;3(5):824. 10.1136/bmjgh-2018-000824 PMC6203064 30398222

[78] Bhana A , Abas MA , Kelly J , van Pinxteren M , Mudekunye LA , Pantelic M . Mental health interventions for adolescents living with and affected by HIV in low‐and middle‐income countries – a systematic review. BJPsych Open. 2020;6(e104):1–15. 10.1192/bjo.2020.67 PMC7488323 32886056

[79] Skeen SA , Sherr L , Croome N , Gandhi N , Roberts KJ , Macedo A , et al. Interventions to improve psychosocial well‐being for children affected by HIV and AIDS: a systematic review. Vulnerable Child Youth Stud. 2017;12(2):91–116. 29085436 10.1080/17450128.2016.1276656 PMC5659734

[80] Willis N , Milanzi A , Mawodzeke M , Dziwa C , Armstrong A , Yekeye I , et al. Effectiveness of community adolescent treatment supporters (CATS) interventions in improving linkage and retention in care, adherence to ART and psychosocial well‐being: a randomised trial among adolescents living with HIV in rural Zimbabwe. BMC Public Health. 2019;19(1):117. 30691425 10.1186/s12889-019-6447-4 PMC6348677

[81] Betancourt TS , Ng LC , Kirk CM , Munyanah M , Mushashi C , Ingabire C , et al. Family‐based prevention of mental health problems in children affected by HIV and AIDS: An open trial. AIDS. 2014;28(3):S359–68. 24991909 10.1097/QAD.0000000000000336 PMC4315615

[82] Shamu S , Abrahams N , Temmerman M , Musekiwa A , Zarowsky C . A systematic review of African studies on intimate partner violence against pregnant women: Prevalence and risk factors. PLoS One. 2011;6:e17591. 21408120 10.1371/journal.pone.0017591 PMC3050907

[83] Malan M , Spedding MF , Sorsdahl K . The prevalence and predictors of intimate partner violence among pregnant women attending a midwife and obstetrics unit in the Western Cape. Glob Ment Health. 2018;5:e18. 10.1017/gmh.2018.9 PMC5981656 29868238

[84] Sheikh MA , Abelsen B , Olsen JA . Differential recall bias, intermediate confounding, and mediation analysis in life course epidemiology: An analytic framework with empirical example. Front Psychol. 2016;7:1828. 27933010 10.3389/fpsyg.2016.01828 PMC5120115

